# Genomic Features of Organ-Specific Metastases in Lung Adenocarcinoma

**DOI:** 10.3389/fonc.2022.908759

**Published:** 2022-07-14

**Authors:** Alei Feng, Yanjun Li, Guangxu Li, Yu Wang, Qiang Wen, Zhe Yang, Kaihua Tian, Hongying Lv, Lijie Guo, Shanshan Zhang, Xiaoyan Liu, Da Jiang

**Affiliations:** ^1^ Tumor Research and Therapy Center, Shandong Provincial Hospital Affiliated to Shandong First Medical University, Jinan, China; ^2^ Shandong Qidu Pharmaceutical Co. Ltd., Shandong Provincial Key Laboratory of Neuroprotective Drugs, Zibo, China; ^3^ Department of Thoracic Surgery, The Second People’s Hospital of Dezhou, Dezhou, China; ^4^ Department of Thoracic Surgery, The Affiliated Hospital of Qingdao University, Qingdao, China; ^5^ Shanghai OrigiMed Co., Ltd, Shanghai, China; ^6^ Medical Oncology, Fourth Hospital of Hebei Medical University, Shijiazhuang, China

**Keywords:** lung adenocarcinoma, bone metastases, liver metastases, brain metastases, next-generation sequencing

## Abstract

**Background:**

The genomic features of cancer cells may confer the metastatic ability of lung adenocarcinoma (LUAD) to metastasize to specific organs. We aimed to identify the differences in genomic alterations between patients with primary LUAD with and without metastases and to elucidate the metastatic biology that may help developing biomarker-directed therapies for advanced or metastatic disease.

**Methods:**

A retrospective cohort of 497 patients with LUAD including 388 primary tumors (PR), 53 bone metastases (MT-bone), 30 liver metastases (MT-liver), and 26 brain metastases (MT-brain) was tested for genomic alterations by a next-generation sequencing assay.

**Results:**

The *EGFR*, *TP53*, *TERT*, *LRP1B*, *CDKN2A*, *ERBB2*, *ALK*, and *KMT2C* genes had a high frequency of mutations, and the mutations were shared by PR and metastases groups. *TP53* and *EGFR* were the most common mutated genes. In comparison with PR, *KRAS*, *STK11*, *ATM*, *NPM1*, and *ROS1* were significantly mutated in MT-brain, and *TP53*, *MYC*, *RSPO2*, *CDKN2a*, and *CDKN2B* were significantly mutated in MT-liver. The frequencies of *TP53*, *CDKN2A*, *MTAP*, *PRKCI*, and *APC* mutations were higher in MT-bone than that in PR. The ERBB, phosphoinositide-3-kinase/protein kinase B (PI3K-AKT), cell cycle, Fibroblast growth factor (FGF), and homologous recombination deficiency signaling pathways were affected in both PR and metastases, and there is higher frequency of mutations in metastases. Moreover, the co-mutations in patients with PR and metastasis were respectively analyzed. In addition, the programmed death ligand 1 (PD-L1) level was obviously related to tumor stage and tumor metastases, and the tumor mutational burden was correlated to clinicopathological features including age, gender, pathological stages, and tumor metastases. *FGFR1*, *KAT6A*, *MYC*, *RAD21*, *TP53*, and *DAXX* were also dramatically correlated to the tumor mutational burden.

**Conclusion:**

Metastases are the most devastating stage of tumors and the main cause of cancer-related deaths. Our results provided a clinically relevant view of the tumor-intrinsic mutational landscape of patients with metastatic LUAD.

## Introduction

Metastasis to distant organs remains the leading cause of cancer-related death (1). Metastasis is a dynamic process involving the dissemination of cancer cell from their primary site to a distant organ and the subsequent colonization of cancer cells at that distant site (1). Most cancers have specific metastatic patterns: a phenomenon known as “organotropism’’ or “organ-specific metastasis” (2). For lung cancer, the most common sites of metastasis are the contralateral lung, brain, bone, and liver (3, 4).

Lung cancer is the most common cancer in the world and the leading cause of cancer death (5), which has the highest morbidity and mortality rates in the world (6). For patients with metastatic lung cancer, the overall survival of 5-year was less than 5% until the last decade. Clinical observations suggested that metastatic sites may predict prognosis (3). The outcomes of the liver and bone metastases were inferior to brain metastasis (3). Lung adenocarcinoma (LUAD) is the most common subtype of lung cancer (7), preferentially metastasizes to the brain, liver, contralateral lung, bone, and adrenal system (3). Although targeted therapies have significantly improved the treatment of patients with LUAD for the past few years, the prognosis is still poor (8), and more investigation on the additional therapeutic targets for LUAD is needed.

Targeted therapy and immunotherapy often require genomic alteration or their derivatives to serve as biomarkers for the identification of appropriate patients (9). All malignant tumor is the result of genetic variation, and the development of tumor is a process of accumulation of genetic and epigenetic changes (10). Next-generation sequencing (NGS) of the primary tumor samples has been widely acted as a practical method for identifying genetic variation in patients with lung cancer (11). In general, metastatic cancers carry mutations similar to those of the primary cancer, but additional mutations occur after transformation (12). NGS has been widely used to identify genetic variation between groups of the primary tumor samples and metastases in LUAD, but the association of cancer-intrinsic mutational status with organ-specific metastases in LUAD remains unclear. Elucidating the metastatic biology of lung cancer may help developing biomarker-directed therapies and improving treatment strategies of advanced or metastatic disease.

To evaluate the genomic features of cancer cells that may confer the metastatic ability to specific organs, we investigated the association of cancer-intrinsic mutational status with organs of metastases. Understanding the genomic profiles of non-metastatic and metastatic tumors at different sites could help guide the treatment of LUAD and future drug development.

## Material and Methods

### Patients

A total of 497 Chinese patients with LUAD were selected from 959 patients for this study, whose tumor tissue and matched blood specimens were collected by authors from patients admitted to the Shandong Provincial Hospital Affiliated to Shandong First Medical University and Fourth Hospital of Hebei Medical University during December 2017 to March 2020. From the 497 eligible patients with LUAD, 388 patients failed to detect any tumor out of the primary lung adenocarcinoma (PR) and 109 patients had metastasis, including 30 patients with liver metastases (MT-liver), 26 patients with brain metastases (MT-brain), and 53 patients with bone metastases (MT-bone). This study was approved by the Institution Review Board according to the Declaration of Helsinki and obtained the informed consent from all enrolled patients.

### Sample Preparation

The formalin-fixed paraffin-embedded (FFPE) tumor samples and matched blood samples were retrieved from the accredited clinical hospitals. The NGS results of these two (tissue and blood) sampling methods are not completely consistent, and when used in clinical practice, they can be conditionally collected simultaneously as a mutual supplement to make the genetic information of tumor cells obtained by analysis more complete. The diagnosis of the histologic subtyping was affirmed through independent pathologists from OrigiMed (Shanghai). The percentage of tumor cells in each sample was 20% or more, and at least 50 ng of tumor tissue DNA was extracted for subsequent genetic analysis.

### Targeted NGS and Genetic Analysis

Genomic profiling was carried out using a targeted panel of 450+ cancer-related genes (Yuansu, Origimed Inc.) (13, 14). The FFPE samples and matched blood samples were obtained for genetic alteration testing. Tumor mutation burden (TMB) was defined as somatic mutation of genomic detection, including coding base substitutions and indel mutation per megabase (muts/Mb). We defined TMB ≥ 10 muts/Mb as TMB-High and <10 muts/Mb as TMB-Low (15).

### PD-L1 Staining

The PD-L1 staining was performed as previously described (16). The FFPE samples were stained using anti–PD-L1 antibody (Abcam, Cambridge, UK), and the percentage of positive PD-L1 staining cells was counted. Positive membrane staining of 1% of tumor cells or tumor-infiltrating immune cells was defined as positive for PD-L1.

### Statistical Analysis

The Fisher’s exact test was used to analyze the relationship between TMB or PD-L1 expression and clinical indexes. Wilcoxon test and *T*-test were performed to analyzed the genes correlated to TMB. The Fisher’s exact test and the Chi-square test were used to compare gene-level mutation frequency between PR and MT-bone, MT-liver, or MT-brain. A *p*-value ≤ 0.05 was recognized statistically significant.

## Results

### Patient Characteristics

A total of 497 patients with LUAD were investigated in this study. Among them, 388 (78%) samples were diagnosed as primary tumors, and 53 (10.7%), 30 (6.0%), and 26 (5.2%) samples were metastases in bone, liver, and brain, respectively. In patients with primary LUAD, there were 181 (46.6%) stage I, 95 (24.4%) stage IV, 67 (17.3%) stage III, and 40 (10.3%) stage II patients, respectively. The median age of patients with primary LUAD was 61, ranging from 32 to 81; and that of patients with bone metastasis was 59, ranging from 33 to 82; patients with liver metastasis was 63.5, ranging from 36 to 85; and patients with liver metastasis was 60, ranging from 26 to 72. Among the 388 patients with primary LUAD, 211 were female and 177 were male; among the 53 patients with bone metastasis, 23 were female and 30 were male; among the 30 patients with liver metastasis, 13 were female and 17 were male; and among the 26 patients with brain metastasis, 10 were female and 16 were male. In patients with primary LUAD, 116 patients had smoked and 254 never smoked. Among the patients with bone metastasis, 12 patients had smoked and 29 never smoked; among the patients with liver metastasis, 12 patients had smoked and 14 never smoked; and among the patients with brain metastasis, three had a history of smoking and 17 never smoked. The detailed clinical characteristics of these patients were listed in **Table 1**.

**Table 1:** Clinicopathological profile of patients.

	Primary tumors (n = 388)	Bone metastases (n = 53)	Liver metastases (n = 30)	Brain metastases (n = 26)
**Age**				
Median	61	59	63.5	60
Range	32–81	33–82	36–85	26–72
**Gender**				
Male	177 (45.6%)	30 (56.6%)	17 (56.7%)	16 (61.5%)
Female	211 (54.4%)	23 (43.4%)	13 (43.3%)	10 (38.5%)
**Smoking status**				
Smokers	116 (29.9%)	12 (22.65%)	12 (40.0%)	3 (11.5%)
Non-smokers	254 (65.5%)	29 (54.7%)	14 (46.7%)	17 (65.4%)
NA	18 (4.6%)	12 (22.65%)	4 (13.3%)	6 (23.1%)
**Stage**				
I	182 (46.6%)	0 (0.0%)	0 (0.0%)	0 (0.0%)
II	42 (10.8%)	0 (0.0%)	0 (0.0%)	0 (0.0%)
III	67 (17.3%)	2 (3.8%)	0 (0.0%)	0 (0.0%)
IV	96 (24.7%)	51 (96.2%)	30 (100.0%)	26 (100.0%)
NA	1 (0.3%)	0 (0.0%)	0 (0.0%)	0 (0.0%)
**PD-L1**				
Negative	117 (30.2%)	27 (50.9%)	10 (33.3%)	9 (34.6%)
Positive	26 (6.7%)	6 (11.3%)	5 (16.7%)	6 (23.1%)
NA	245 (63.1%)	20 (37.7%)	15 (50.0%)	11 (42.3%)
**TMB**				
High	67 (17.3%)	8 (15.1%)	6 (20.0%)	13 (50.0%)
Low	294 (75.8%)	39 (73.6%)	23 (76.7%)	13 (50.0%)
NA	27 (6.9%)	6 (11.3%)	1 (3.3%)	0 (0.0%)

### The Mutational Landscape in Primary Tumors and Metastases

**Figure 1:**
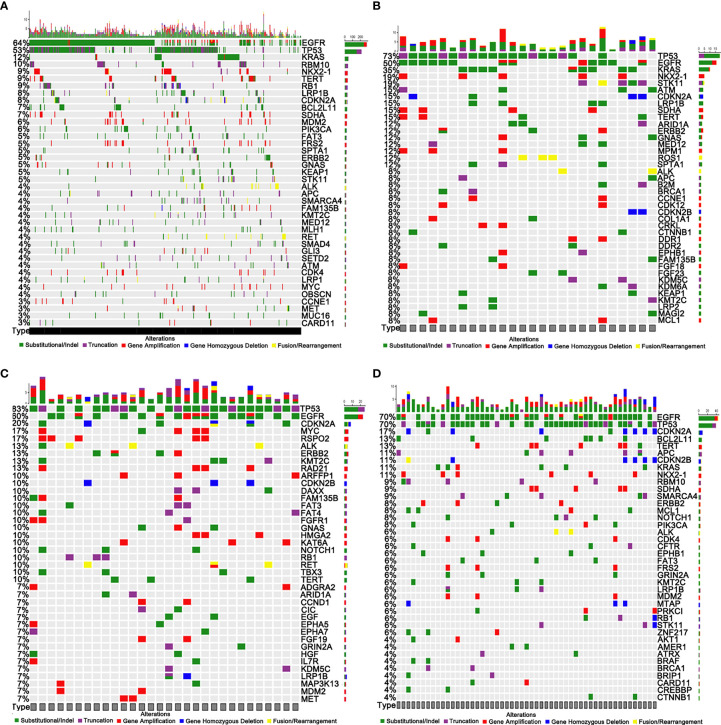
Genomic profiling of somatically altered genes and frequently mutated genes. **(A)** Primary tumors (PR). **(B)** Brain metastases (MT-brain). **(C)** Liver metastases (MT-liver). **(D)** Bone metastases (MT-bone).

A total of 4843 mutations were found, including 209 fusions/rearrangements, 1,202 gene amplifications, 86 gene homozygous deletions, 1,627 substitutions, and 407 truncations, in the entire cohort: primary tumors (PR) and metastases (MT-liver, MT-bone, and MT-brain). The top 40 mutated genes in PR, MT-brain, MT-liver, and MT-bone were demonstrated in **Figures 1A–D**, respectively. The *EGFR*, *TP53*, *TERT*, *LRP1B*, *CDKN2A*, *ERBB2*, *ALK*, and *KMT2C* genes had a high frequency of mutations, and the mutations were shared by PR and metastases (MT-liver, MT-bone and MT-brain). *TP53* and *EGFR* were the most frequently mutated genes, among which *TP53* accounted for 83% of MT-liver and *EGFR* accounted for 70% of MT-bone. Notably, *MAP3K13* mutation was found exclusively in MT-liver.

### Discrepancy of Mutation Frequency Between Primary Tumors and Metastases

**Figure 2:**
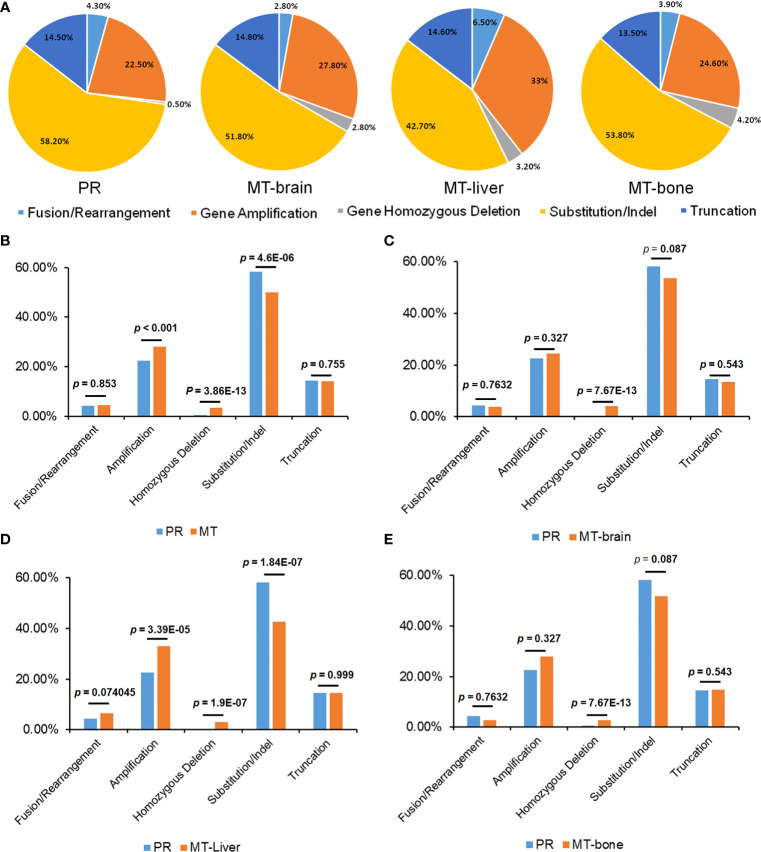
The distribution of mutation types of primary tumors and metastases. **(A)** The percentages of different mutation types in PR and metastases. **(B)** A comparison of the proportions of mutation types between patients with PR and metastasis, **(C)** PR and MT-brain, **(D)** PR and MT-liver, and **(E)** PR and MT-bone.

**Figure 3:**
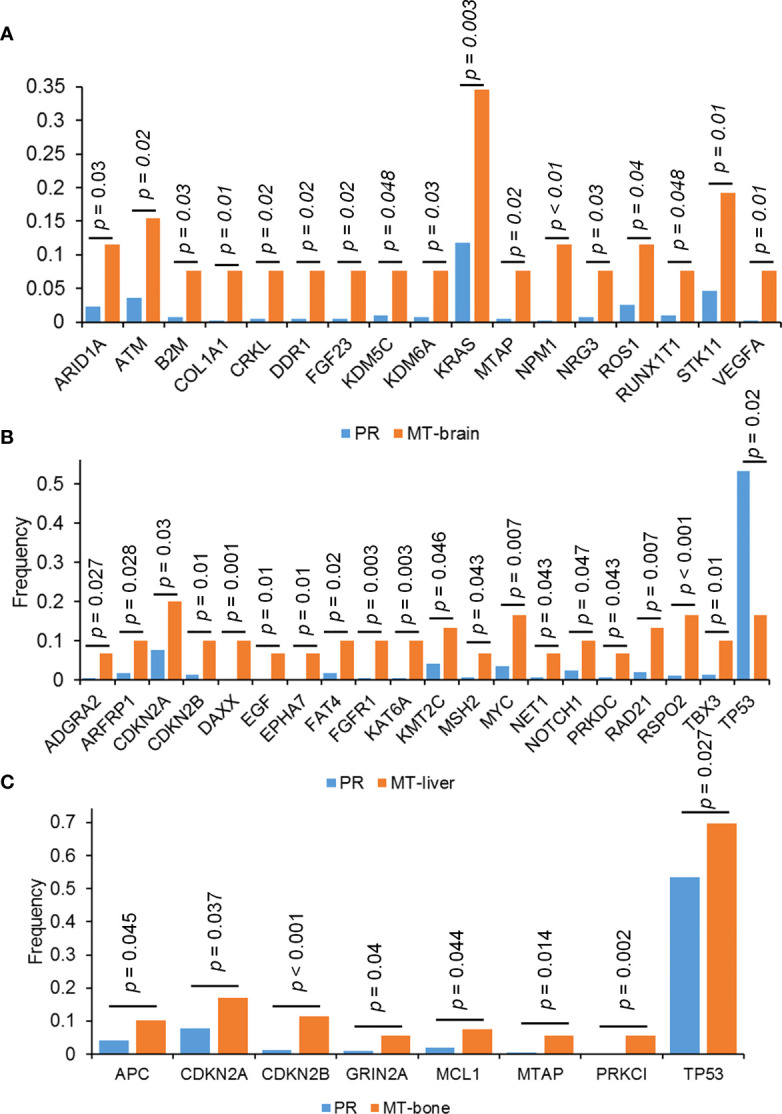
The differences of genomic mutations between PR and metastases. The differences of genomic mutations between **(A)** PR and MT-brain, **(B)** PR and MT-liver, and **(C)** PR and MT-bone.

The frequency of mutations was compared between PR and metastases by using the Fisher’s exact test. In comparison with PR, MT-brain, MT-liver, and MT-bone had more gene amplifications and gene homozygous deletions but fewer substitutions (**Figures 2A–E**). In addition, the frequently mutated genes in PR and metastases were displayed in **Figures 3A–C**. In comparison with PR, the mutations of *ARID1A*, *ATM*, *B2M*, *COL1A1*, *CRKL*, *DDR1*, *KDM5C*, *KDM6A*, *KRAS*, *MTAP*, NPM1, *NRG3*, *ROS1*, *RUNX1T1*, *STK11*, and *VEGFA* were significantly more frequent in MT-brain (**Figure 3A**). The mutation frequencies of *ADGRA2*, *ARFRP1*, *CDKN2B*, *DAXX*, *EGF*, *EPHA7*, *FAT4*, *FGFR1*, *KAT6A*, *KMT2C*, *MSH2*, *MYC*, *NET1*, *NOTCH1*, *PRKDC*, *RAD21*, *RSPO2*, *TBX3*, and *TP53* were significantly different between PR and MT-liver (**Figure 3B**), whereas the mutation frequencies of *APC*, *CDKN2A*, *CDKN2B*, *GRIN2A*, *MCL1*, *MTAP*, *PRKCI*, and *TP53* were significantly different between PR and MT-bone (**Figure 3C**).

**Figure 4:**
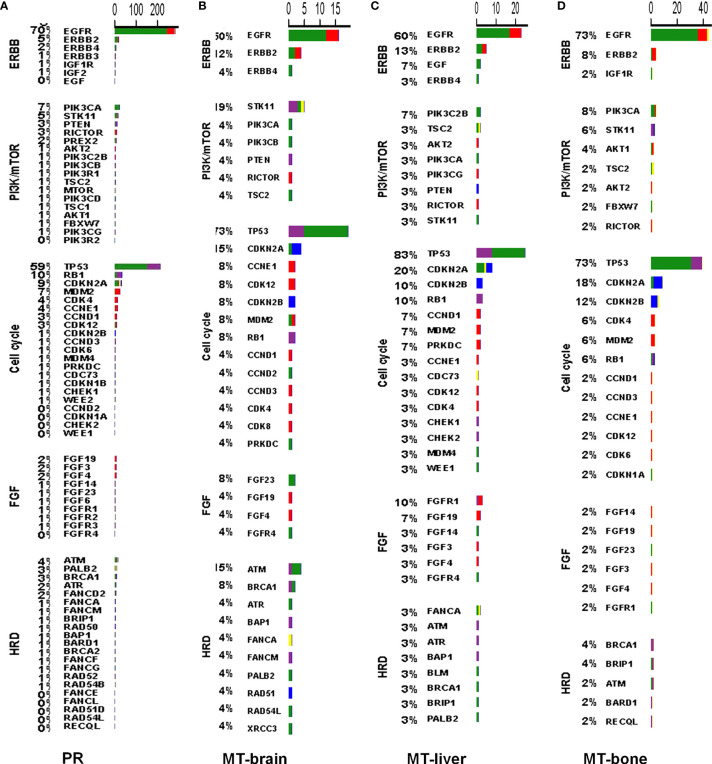
The mutation profiles according to signaling pathways in PR and metastases. The mutation profiles of the top five affected signaling pathways in **(A)** PR, **(B)** MT-brain, **(C)** MT-liver, and **(D)** MT-bone.

To better discover the affected signaling pathways and guide the development of targeted therapy, we classified patients according to the presence and absence of mutation in each signaling pathway. The top five affected signaling pathways were ERBB, PI3K-AKT, cell cycle, FGF, and homologous recombination deficiency in PR and metastatic tumors, with a higher frequency of mutations in patients with metastasis (**Figure 4**).

### Co-Mutation Analysis in Primary Tumors and Metastases

**Figure 5:**
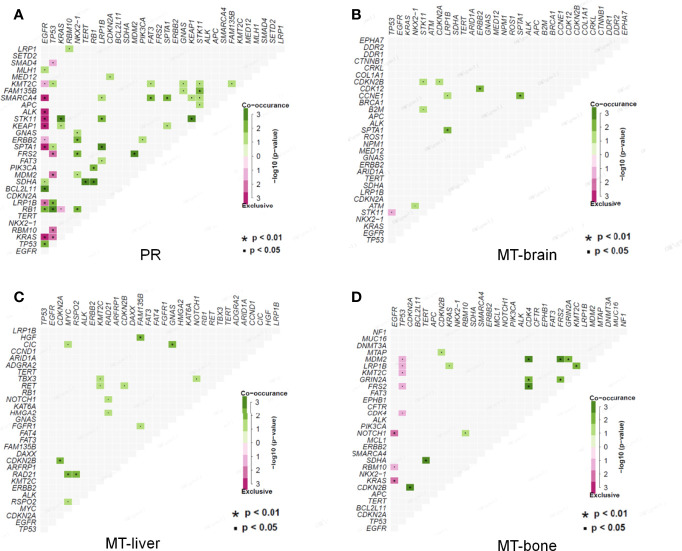
Co-occurrence of genomic alterations in PR and metastases. **(A)** The co-occurrence relationship between genomic mutations in PR is displayed in heatmap. **(B–D)** The co-occurrence relationships between genomic mutations in MT-brain, MT-liver, and MT-bone are shown in corresponding heatmaps, respectively.

Co-mutations could provide information for drug combination therapy and medication instruction. In PR, *EGFR* mutations and *SMARCA4*, *ALK*, *STK11*, *KEAP1*, *SPTA1*, *LRP1B*, and *KRAS* mutations were mutually exclusive, but *EGFR* mutations were found to be co-occurred with *BCL2L11*, *RB1*, and *TP53* mutations. *TP53* mutations were significantly co-occurred with *LRP1B* and *RB1* mutations. *KRAS*, *KEAP1*, and *FAM135B* mutations were co-occurred with *STK11* mutations, and *NKX2-1* mutations were co-occurred with *ERBB2*, *FRS2*, and *RB1* mutations, respectively. *SDHA* mutations were significantly co-occurred with *TERT* and *RB1* mutations. *MDM2* mutations were significantly co-occurred with *FRS2* mutations. *SMARCA4* mutations were co-occurred with *FAT3* and *SPTA1* mutations (**Figure 5A**). In MT-brain, *LRP1B* mutations were significantly co-occurred with *SPTA1* mutations, *ERBB2* mutations were co-occurred with *CDK12* mutations, and *SPTA1* mutations were significantly co-occurred with *CCNE1* mutations (**Figure 5B**). In MT-liver, *CDKN2B* mutations were significantly co-occurred with *CDKN2A* mutations, and *RAD21* mutations were obviously co-occurred with *MYC* and *RSPO2* mutations. *HGF* mutations were co-occurred with *FAM135B* mutations, and *CIC* mutations were co-occurred with *GNAS* mutations (**Figure 5C**). In MT-bone, *MDM2* and *GRIN2A* mutations were both significantly co-occurred with *CDK4*, *FRS2*, and *GRIN2A* mutations, and *CDKN2B* mutations were co-occurred with *CDKN2A* mutations. *SDHA* mutations were co-occurred with *TERT* mutations, and *LRP1B* mutations were co-occurred with *KMT2C* mutations (**Figure 5D**).

### Correlation Between Expression of PD-L1, TMB, and Clinicopathological Characteristics

**Figure 6:**
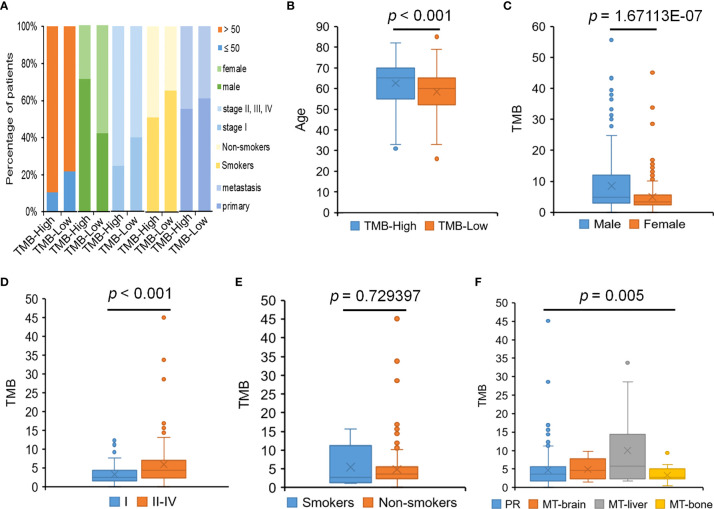
The relationship between TMB and clinical features. **(A)** The percentage of patients with different clinical features in the TMB-High and TMB-Low groups. **(B)** The relationship between TMB and age. **(C)** The relationship between TMB and gender. **(D)** The relationship between TMB and pathological stages. **(E)** The relationship between TMB and smoking history. **(F)** The relationship between TMB and tumor metastasis.

TMB is a promising prognostic biomarker for immunotherapy across multiple cancer types. The median TMB for the cohort was 4.3 muts/Mb, with a range from 0.5 to 55.7. TMB-High was seen in 18.9% of patients, and TMB-Low was seen in 74.2% of patients. Higher percentages of patients had the late tumor stage and tumor metastasis condition, which were also displayed in the TMB-High group, suggesting that these clinical factors might be relate to higher TMB to some degree (**Figure 6A**). We determined the relationship between TMB level and the clinicopathological characteristics of LUAD. The results observed that the level of TMB was obviously correlated to the age, gender, pathological stages, and tumor metastasis (**Figures 6B–F**). TMB was also significantly associated with *APC*, *KRAS*, *NOTCH1*, *SMARCA4*, *STK11*, and *ATRX* mutations (**Table 2**).

**Table 2:** The mutated genes associated with TMB.

Gene	T-testp-value	Wilcox test p-value	Gene	T-testp-value	Wilcox test p-value
*APC*	0.041871	0.004278	KMT2C	0.003101	3.32E−05
*ATRX*	0.029324	0.002635	KRAS	0.01462	8.58E−04
*BRCA1*	0.014862	3.69E−04	LRP1B	2.19E−04	1.96E−08
*CIC*	0.04393	0.001201	LRP2	0.046485	0.010335
*DICER1*	0.04464	0.004705	MAGI2	0.020854	4.43E−04
*EGFR*	9.56E−06	1.05E−04	MUC16	0.002019	4.93E−06
*ERBB4*	0.021124	0.004603	NOTCH1	0.029449	0.002045
*ERRFI1*	0.04559	0.00235	NOTCH2	0.045895	0.002165
*FAM135B*	0.005188	1.26E−04	NTRK2	0.015291	0.004905
*FANCA*	0.049024	2.57E−04	PLCG2	0.041594	0.002491
*FAT3*	0.014584	2.93E−04	POLE	0.00878	8.55E−06
*FAT4*	0.009024	3.19E−04	RB1	0.03535	8.56E−04
*GATA1*	0.016516	9.58E−04	SMARCA4	0.001403	2.43E−06
*GATA3*	0.036069	0.037538	SOX9	0.048958	0.011621
*GLI1*	0.029308	0.008542	SPEN	0.020829	6.66E−04
*GNAS*	0.024932	0.014035	SPTA1	4.57E−04	1.03E−06
*IL7R*	0.027336	8.68E−04	STK11	0.003398	2.95E−06
*KDR*	0.036724	0.009621	TP53	1.40E−06	1.94E−07
*KEAP1*	0.02231	0.00137			

**Figure 7:**
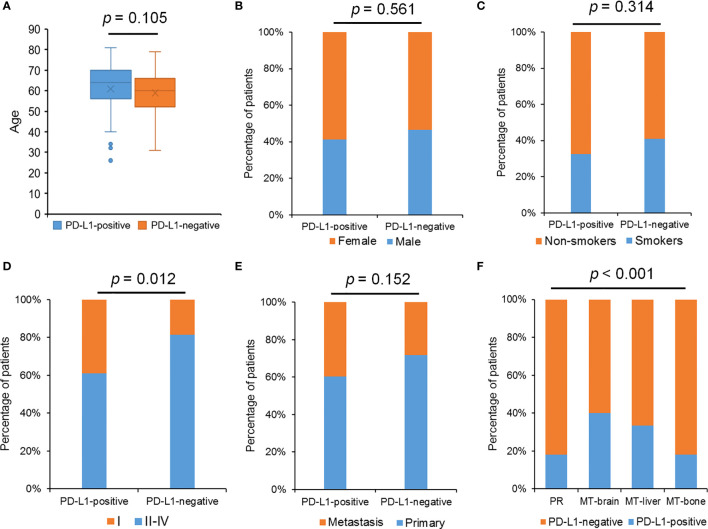
The relationship between PD-L1 expression and clinical features. **(A)** The relationship between PD-L1 expression and age. **(B)** The percentage of patients with different gender in the PD-L1–positive group and the PD-L1–negative group. **(C)** The percentage of patients with smoking history in the PD-L1–positive group and the PD-L1–negative group. **(D)** The percentage of patients with different pathological stages in the PD-L1–positive group and the PD-L1–negative group. **(E)** The percentage of patients with different tumor status in the PD-L1–positive group and the PD-L1–negative group. **(F)** The percentage of patients with different tumor metastasis status in the PD-L1–positive group and the PD-L1–negative group. The Chi-square test was used to detect *p*-values, and *p*-value ≤ 0.05 was recognized statistically significant.

Meanwhile, the relationships between the PD-L1 level and the clinicopathological features of LUAD were also analyzed. A total of 206 patients with LUAD enrolled in our study had PD-L1 staining results, and the proportion of PD-L1–positive cases accounted for 79.1%. The 206 patients included 15 patients with MT-brain (PD-L1 negative, 6; PD-L1 positive, 9), 33 patients with MT-bone (PD-L1 negative, 6; PD-L1 positive, 27), and 15 patients with MT-liver (PD-L1 negative, 5; PD-L1 positive, 10). We observed that PD-L1 expression was dramatically related to tumor metastases and pathological stages, whereas there was no significant relationship between the PD-L1 level and the other clinicopathological indexes including age, gender, and smoking history (**Figure 7**).

## Discussion

Most lung cancers have already metastasized at the time of initial diagnosis, and the survival rate of 5-year is poor (17). LUAD accounts for nearly 61% of the pathological subtypes of lung cancer, so it is of great research value to improve the survival rate of LUAD (18). Although more attention has been paid on LUAD gene sequencing, the genetic profile and the underlying mechanisms of metastatic cancer progression are still poorly understood. We hope that the discovery of the differences in genomic landscape between patients with primary LUAD with and without metastases in our study could provide guidance for the treatment and future drug development of LUAD.

Our study found that the frequently mutated genes including *EGFR*, *TP53*, *KRAS*, *TERT*, *LRP1B*, *CDKN2A*, *ERBB2*, *ALK*, and *KMT2C* were shared by PR and metastases. We found that *EGFR* mutations were frequently mutated in PR (64%), MT-brain (50%), MT-liver (50%), and MT-bone (70%). The results was consistent with a previous study reporting that *EGFR* was the most frequently shared driver gene, which accounted for more than 40%–50% of the whole LUAD population (19). *KRAS* mutations have been observed to be related to poor prognosis in resected lung cancer, lack of survival benefit from adjuvant chemotherapy, and resistance to erlotinib or gefitinib (20–22). The results in the study of Pao et al. have indicated that, by determining the mutant status of EGFR and KRAS, treatment decisions for the use of these kinase inhibitors can be improved (22). Gefitinib/erlotinib targeting *EGFR* mutations and crizotinib targeting *ALK* translocations have shown clinical benefit and approved for clinical use (23). telomerase reverse transcriptase (TERT) inhibition has been acted as a promising therapeutic strategy for LUAD (24, 25). These mutated genes of LUAD may serve as potential targets, providing more possibilities and strategies for the treatment of LUAD.

Considering the spatial heterogeneity of the tumor, the genetic mutation status of a few tumor cells in the primary site may not represent the mutation status of distant metastasis (26). *ARID1A*, *ATM*, *B2M*, *COL1A1*, *CRKL*, *DDR1*, *KDM5C*, *KDM6A*, *KRAS*, *MTAP*, *NPM1*, *NRG3*, *ROS1*, *RUNX1T1*, *STK11*, and *VEGFA* were significantly mutated in the 26 patients with brain metastases in our cohort, compared with PR. Shih et al. have shown that the most frequently mutated genes (*MYC*, *TERT*, *MDM2*, *CDK4*, *CCND1*, and *NKX2–2*) in both the brain metastasis–LUAD and The Cancer Genome Atlas (TCGA)-LUAD cohorts (27). A previous study revealed that more *ARID1A* mutations were observed in MT-brain than that in PR (28). The deficiency of MTAP to predict better treatment response in patients with advanced LUAD who receive early pemetrexed platin chemotherapy and bevacizumab (29). *KRAS* mutation was significantly more common in MT-brain than described for extracranial tumor manifestations (30). Whether the newly identified genes highly mutated in both MT-brain and PR can be used as predictive biomarkers and provide more guidance for the treatment of MT-brain remains to be validated. The mutation frequencies of *ADGRA2*, *ARFRP1*, *CDKN2B*, *DAXX*, *EGF*, *EPHA7*, *FAT4*, *FGFR1*, *KAT6A*, *KMT2C*, *MSH2*, *MYC*, *NET1*, *NOTCH1*, *PRKDC*, *RAD21*, *RSPO2*, *TBX3*, and *TP53* were significantly different between PR and MT-liver. Emerging evidence supported that *TP53* mutations augmented the metastatic potential of tumors (31). The underlying mechanism might be correlated to chromosomal instability or drug resistance but still remained to be further elucidated (32). In a study reported by Liao et al., in 2018, *APC* was only mutated in PR but not in MT-brain, whereas our study showed that *APC* was mutated not only in PR but also in MT-brain and MT-bone (33). Our results indicated that the mutation frequencies of *APC*, *CDKN2A*, *CDKN2B*, *GRIN2A*, *MCL1*, *MTAP*, *PRKCI*, and *TP53* were significantly different between PR and MT-bone. *CDKN2A*/*B* were more abundant across all three metastatic cohorts. The mutations of *CDKN2A/B* were frequently involved in genomic deletions. *CDKN2A*/*B* are frequently mutated and tested in various tumors. Several studies indicated *CDKN2A/B* deleted or mutated patients can benefit from CDK4/6 inhibitors (34–37). In a phase II clinical trial (NCT01291017), advanced non–small cell lung cancer patients with wild-type *RB* and inactive *CDKN2A* can benefit from palbociclib treatment. Eight of the 16 patients have achieved stable disease for more than 4 months. Emergence of new genes with significantly higher mutation frequency in each organ metastasis compared with the primary tumors suggests that the different immunotherapeutic responses of each organ metastasis to highly mutated tumors and their sensitivity to PARP inhibitors may be a potential treatment for a specific metastatic organ.

In the past, it is generally accepted that lung cancer drive gene mutations were mutually exclusive (38). With the development of gene detection technology, cases of co-existing driver gene mutations were explored. In PR, *KRAS*, *KEAP1*, and *FAM135B* mutations were co-occurred with *STK11* mutations. This finding was similar to previously result that co-mutations of both *STK11* and *KEAP1* were a strong determinant of unfavorable prognosis with currently available therapies (39). *NKX2-1* mutation was co-occurred with *ERBB2*, *FRS2*, and *RB1* mutations, which was different from a previous study reported a co-mutation of *NKX2-1* and *NEKB1A* in PR (9). Patients with metastasis had different co-mutation profiles from PR. In MT-brain, *LRP1B* mutation was significantly co-occurred with *SPTA1* mutation, and *ERBB2* mutation was co-occurred with *CDK12* mutation. *RAD21* mutation was obviously co-occurred with *MYC* and *RSPO2* mutations in MT-liver. In MT-bone, *MDM2* mutation was significantly co-occurred with *CDK4* mutation, and *LRP1B* mutation was co-occurred with *KMT2C* mutation. Previous studies have shown that co-mutations of *TP53* and *KRAS* can function as potential biomarkers for immune checkpoint blockade in lung cancer (40) and that co-mutations of *KRAS* and *TP53* could identify long-term responders to first-line palliative treatment with pembrolizumab from patients with LUAD with high PD-L1 level (41). In our study, we drafted the co-mutation profiles of LUAD with different site of metastasis, which might provide potential biomarkers for immune checkpoint blockade in LUAD with different organ metastases.

In conclusion, we analyzed the mutational profiles that represent the tumor-intrinsic factors of LUAD metastases. Metastatic cancer is a highly devastating disease. The development of novel systemic treatments of metastatic cancer depends on the insight into the therapeutic implications of metastatic heterogeneity. Our findings on genomic characterization of PR and metastasis provide a viable strategy for discovering potential pathways to prevent and treat metastatic LUAD.

## Data Availability Statement

The raw data supporting the conclusions of this article will be made available by the authors, without undue reservation.

## Ethics Statement

The studies involving human participants were reviewed and approved by Fourth Hospital of Hebei Medical University. The patients/participants provided their written informed consent to participate in this study.

## Author Contributions

AF and DJ contributed to conception and design of the study. LG, SZ, and XL performed the statistical analysis. AF, YL, GL, YW, QW, ZY, KT, HL, and DJ wrote the manuscript. All authors contributed to manuscript revision, read, and approved the submitted version.

## Funding

This study was supported in part by the Natural Science Foundation of Shandong (Grant Nos. ZR2020MH229 and ZR2020QH200), the Radiation Oncology Translational Medicine Foundation for Scientific Research of Betthune (Grant No. flzh202123), and the special foundation for CSCO Cancer Research (Grant Nos. Y-QL2019-0149 and Y-2019AZMS-0522).

## Conflict of Interest

Author AF was employed by Shandong Qidu Pharmaceutical Co. Ltd and LG, SZ and XL are employees of OrigiMed.

The remaining authors declare that the research was conducted in the absence of any commercial or financial relationships.

## Publisher’s Note

All claims expressed in this article are solely those of the authors and do not necessarily represent those of their affiliated organizations, or those of the publisher, the editors and the reviewers. Any product that may be evaluated in this article, or claim that may be made by its manufacturer, is not guaranteed or endorsed by the publisher.
